# Aberrant Meiotic Prophase I Leads to Genic Male Sterility in the Novel TE5A Mutant of *Brassica napus*

**DOI:** 10.1038/srep33955

**Published:** 2016-09-27

**Authors:** Xiaohong Yan, Xinhua Zeng, Shasha Wang, Keqi Li, Rong Yuan, Hongfei Gao, Junling Luo, Fang Liu, Yuhua Wu, Yunjing Li, Li Zhu, Gang Wu

**Affiliations:** 1 Oil Crops Research Institute of the Chinese Academy of Agricultural Sciences/Key Laboratory of Biology and Genetic Improvement of Oil Crops, Ministry of Agriculture, Wuhan 430062, China

## Abstract

Genic male sterility (GMS) has already been extensively utilized for hybrid rapeseed production. TE5A is a novel thermo-sensitive dominant GMS line in *Brassica napus*, however, its mechanisms of GMS remain largely unclear. Histological and Transmission electron microscopy (TEM) analyses of anthers showed that the male gamete development of TE5A was arrested at meiosis prophase I. EdU uptake of S-phase meiocytes revealed that the TE5A mutant could accomplish DNA replication, however, chromosomal and fluorescence *in situ* hybridization (FISH) analyses of TE5A showed that homologous chromosomes could not pair, synapse, condense and form bivalents. We then analyzed the transcriptome differences between young floral buds of sterile plants and its near-isogenic fertile plants through RNA-Seq. A total of 3,841 differentially expressed genes (DEGs) were obtained, some of which were associated with homologous chromosome behavior and cell cycle control during meiosis. Dynamic expression changes of selected candidate DEGs were then analyzed at different anther developmental stages. The present study not only demonstrated that the TE5A mutant had defects in meiotic prophase I via detailed cytological analysis, but also provided a global insight into GMS-associated DEGs and elucidated the mechanisms of GMS in TE5A through RNA-Seq.

Both cytoplasmic male sterility (CMS) and genic male sterility (GMS) are common pollination control systems for hybrid rapeseed production^1^,^2^,^3^. However, GMS, which is considered as an efficient alternative to the CMS system, presents some merits, including stable and complete male sterility, ease of transfer of male sterility genes, and rich sources of cytoplasm that avoids the possible risk of utilizing a single cytoplasmic source^4^. To date, various double low GMS lines have been comprehensively exploited to develop heterotic hybrids^5^,^6^,^7^,^8^,^9^,^10^,^11^. S45 AB, as a recessive GMS line derived from a spontaneous mutant of the canola variety, Oro, is widely used for rapeseed heterosis in China^12^,^13^. GMS line Rs1046AB is derived from a natural mutation of Yi3A^14^. TE5A is a novel thermo-sensitive dominant GMS line that originated from the spontaneous mutation of the *B. napus* inbred line TE5, which is controlled by only one dominant gene and serves as a promising system for the development of hybrid cultivars^15^. However, the molecular mechanisms underlying the control of these GMS lines remain obscure.

In the developmental process of gametogenesis, some cells in the anther, firstly, differentiate into archesporial cells, which produce the primary sporogenous cells. Then the primary sporogenous cells give rise to pollen mother cells (PMCs) and primary parietal cells^16^. Finally, primary parietal cells lead to the tapetum, endothecium, and the middle layer of the anther, and meiosis occurs in PMCs^16^. Meiosis, which is related to gamete development that produces four uniform haploid spores from one diploid PMC, plays a major role in the sexual reproduction process of organisms. During meiosis, two consecutive chromosome divisions follow a single DNA replication^17^. In meiosis I, a coordinated chain of events in the extended prophase I results in pairing, synapsis of homologous chromosomes and recombination of non-sister chromatids in each bivalent. Chromosome number is cut in half in anaphase I owing to the separation of homologous chromosomes^18^. During meiosis II, sister chromatids segregate, following a similar mechanism to that observed in mitosis (equational division). The proper partitioning of genetic material during cell division requires the precise orchestration of cell cycle events^19^.

To produce haploid spores, meiocytes must succeed in entering meiosis I, pass through the transition of the meiosis I to meiosis II, and exit meiosis II. Some errors in these processes are usual and can result in parthenogenesis, teratoma generation, or the production of 2n gametes without a reduction in the number of chromosomes^20^,^21^. The complexes of cyclins and cyclin-dependent kinases (Cdks) play an essential role in the control of the cell cycle. In addition, the activity of Cdk-cyclin complexes regulate the progression of both the meiotic and mitotic cell cycles at determined checkpoints^22^,^23^. Little is known about the control of the plant meiosis. The maize *elongate* mutant, generates 2n female gametes as a result of the failure in female meiosis II, although the related gene has not been identified to date^24^. In the *Arabidopsis thaliana tam-1* mutant, only one replacement of an amino acid (Thr283Ile) disorders the cell cycle control associated with male meiosis, resulting in a failure to enter meiosis II, thus leading to the generation of dyad and diploid spores, thereby suggesting that *CYCA1;2/TAM* plays a crucial role in the transition from meiosis I to meiosis II^25^,^26^. Moreover, *osd1* mutants are unable to enter meiosis II during male and female meioses, leading to the generation of functional diploid gametes and polyploid progeny^27^.

In the present study, cytological analysis indicated that the male development in the novel TE5A GMS line was arrested at meiosis prophase I, as shown by its inability to undergo pairing, recombination and synapsis, and eventually, the PMCs degenerated, thereby giving rise to a relatively stable and complete GMS phenotype. Anther and pollen development are precisely regulated by several external and internal cues, and these processes are crucial for male reproduction. Besides the essential genes in the male developmental network, several plant hormones, such as jasmonic acids, ethylene, auxin, gibberellins, and cytokinins, have an affect on male fertility^28^,^29^,^30^,^31^,^32^, and various biosynthetic phytohormones or signaling mutants of plant hormones have reduced male fertility. In the present study, to elucidate the underlying molecular mechanism of the TE5A GMS that is associated with abnormal meiosis prophase I phase, we used young floral buds (0.5–1.0 mm in diameter, at meiotic prophase I stage) of sterile plants and fertile plants of the BC_3_ population derived from the successive backcrossing of heterozygous TE5A sterile plants with the maintainer line GY12 to perform a genome-wide high-throughput transcriptome sequencing analysis (RNA-Seq) to identify differentially expressed genes (DEGs) participating in the control of fertility.

## Results

### Selection of fertile and sterile plants with the closest genic background

**Figure 1:**
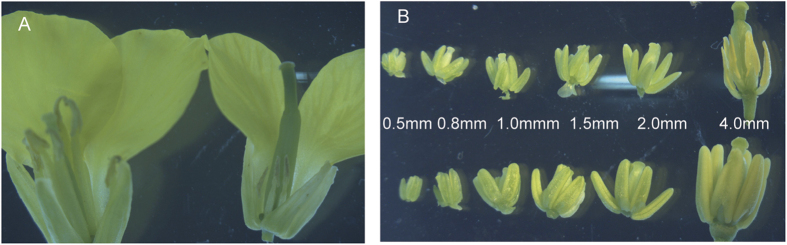
Comparison of floral morphology between sterile and fertile plants. (**A**) young buds of sterile and fertile plants (left, fertile; right, sterile); (**B**) anthers corresponding to different lengths of young buds (0.5, 0.8, 1.0, 1.5, 2.0, and 4.0 mm; upper, sterile; down, fertile).

TE5A is a novel DGMS line that originated from a spontaneous mutant of the inbred line TE5. It exhibits ecotypic temperature sensitivity, it is fertile at low temperatures, and transforms to a completely sterile phenotype at temperatures of >20 °C during florescence. Therefore, the critical temperature controlling the fertility of TE5A was 20 °C. To acquire plants with the most similar genetic background, one heterozygous TE5A sterile plant was utilized as the female parent and crossed with the temporary maintainer GY12, resulting in an F_1_ population that presented fertility segregation. Sterile F_1_ plants were backcrossed to GY12 to produce the BC_1_ population. The BC_3_ population was then obtained by backcrossing sterile plants of the BC_2_ population with GY12. Its fertility was determined during flowering when the temperature was >20 °C. Sterile plants and fertile plants with a 1:1 fertility segregation ratio were derived from the BC_3_ population of the same genic background besides the sterile gene locus residing in sterile plants. At the same temperature (>20 °C), the fertile plants showed normal floral morphology and architecture, whereas sterile plants presented defective male floral organs (abnormal anthers, no pollen was produced, and stamens showing a reduction in size), and had normal flowers in other respects (Fig. 1A). During the early stages of stamen development, the sterile plants showed full and well developed stamens compared to that observed in the wild plants. Alterations were observed at the later stamen stage, stamens of fertile plants exhibited well-developed, yellow anthers, whereas sterile plants presented withered anthers with no pollen (Fig. 1B).

### Phenotypic characteristics of TE5A male gamete development

Male gamete development is initiated within the anther, where sporogenous initial cells, also called PMCs, enter the meiotic cycle to form a tetrad of cells that eventually differentiate into reproductive tissues. Mitotic divisions and microspore maturation follows meiosis of the PMCs, leading to mature pollen grains. Highly specialized anther tissues acquire reproductive (male gamete formation) or non-reproductive (such as stomium for dehiscence, the tapetum for support) functions. Microspore and tapetum development are both essential to male fertility, and numerous studies on male sterile mutants have been reported^33^,^34^,^35^,^36^,^37^,^38^,^39^.

**Figure 2:**
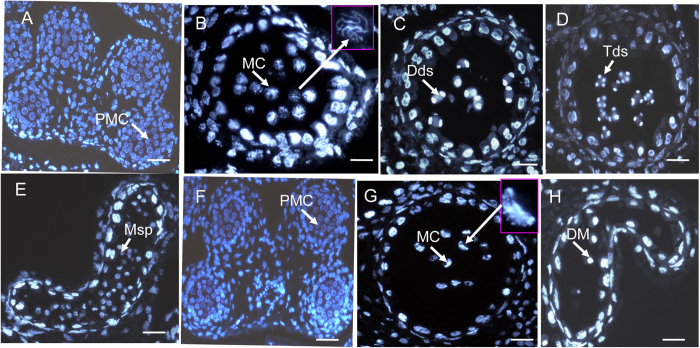
DAPI-stained cross-sections of anthers in both the wild-type (**A–E**) and the TE5A mutant (**F–H**). (**A,F**), the early PMC stage; (**B,G**), the meiosis stage; (**C**) the dyad stage; (**D**) the tetrad stage; (**E**) the microspore stage; (**H**) the degenerating meiocyte stage. PMC, pollen mother cell; MC, meiotic cell; Dds, dyads; Tds, tetrads; Ms, microspore; DM, degenerating meiocyte. Scale bars = 50 μm.

To determine which step of male development was disrupted in the TE5A mutant, paraffin-cross sections of anthers from different developmental stages were analyzed through DAPI staining (Fig. 2). There are 14 well-ordered stages for anther development which is based on the morphological characteristics of *Arabidopsis*^40^. In the TE5A mutant, the early anther PMC development stage appeared normal compared to the wild-type (Fig. 2A,F). However, several differences in anther development between the wild-type and the TE5A mutant occurred after the early PMC stage. Alterations initially appeared at the MC stage, chromosomes were normally condensed into thread-like structures during wild-type meiosis, corresponding to the zygotene phase of prophase I (Fig. 2B), whereas chromosomes of the mutant were aberrantly condensed into crescent-like structure (Fig. 2G). In the later stages, in the wild-type, dyads were generated, indicating meiosis I had been completed (Fig. 2C), which was subsequently followed by the formation of tetrads (Fig. 2D) and microspores (Fig. 2E). In contrast, in the TE5A mutant, chromosomes were further condensed and eventually degenerated, and thus no dyads or tetrads were generated, thereby suggesting that the TE5A mutant failed in accomplishing meiosis I and entering meiosis II, and PMCs were arrested at meiosis I (Fig. 2H).

**Figure 3:**
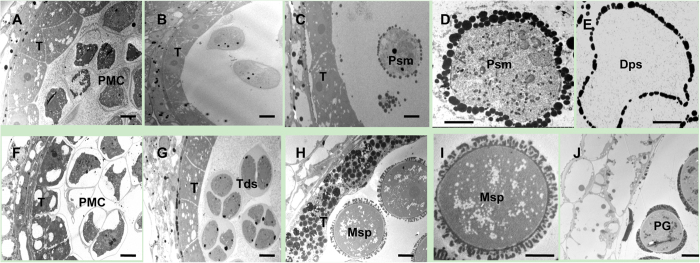
TEM micrographs of the anthers from the wild-type (**F–J**) and the TE5A mutant (**A–E).** (**A,F**), early meiotic PMC stage with no detectable differences between the TE5A mutant (**A**) and the wild type (**F**); (**B,G**), the tetrad stage, in the wild type, the PMCs could complete meiosis I and enter the tetrad stage with tetrad formation (**G**), whereas no dyads or tetrads were observed in the TE5A mutant (**B**); (**C,D,H,I**), the microspore stage, microspores were released from the tetrads and covered with an microspore wall in the wild-type (**H,I**). However, the PMCs of the mutant did not undergo meiosis and developed into pseudo-microspores surrounded by a layer of grains, instead of a typical microspore wall (**C,D**); (**E,J**), the pollen grain stage, the wild-type microspores developed into mature pollen grains with the normal exine wall (**J**), whereas pseudo-microspores of the TE5A mutant were degenerated, resulting in an empty locule. T, tapetum; PMC, pollen mother cell; Tds, tetrad; Msp, microspore; Psm, pseudo-microspore; Dps, degenerating pseudo-microspore; PG: pollen grain; Scale bars = 10 μm.

Analysis of paraffin-cross sections of anther sections had indicated that the early detectable defect in the TE5A mutant involved the disruption of meiosis I, which then led to a failure in dyad or tetrad generation in the anther. To further understand the abnormalities in the mutant, TEM was used to compare the differences in anther development between the mutant and wild-type plants. First, tapetal development during meiosis was normal in the TE5A mutant compared to that in the wild-type (Fig. 3). For male mate development, similar to the observations using paraffin-cross sections, no detectable differences in the early PMC stage were observed between the TE5A mutant and the wild-type (Fig. 3A,F). However, some differences in male mate development after the early PMC stage were observed between the mutant and the wild-type. In the wild-type, the PMCs completed meiosis I and entered the tetrad stage (Fig. 3G). However, at this stage, no dyads or tetrads were observed in the TE5A mutant, and only PMCs were observed (Fig. 3B). Subsequently, the wild-type microspores were released from the tetrads and were covered with a microspore wall (Fig. 3H,I), and in contrast to that observed in the wild-type, the PMCs of the mutant did not accomplish meiosis I and developed into pseudo-microspores that were surrounded by a layer of grains, instead of the a typical microspore wall (Fig. 3C,D). Finally, the wild-type microspores underwent mitosis and developed into mature pollen grains with the normal exine wall (Fig. 3J), whereas the pseudo-microspores of the TE5A mutant degenerated and instead showed an empty locule (Fig. 3E). TEM analysis suggested that the abnormal anther development in the TE5A mutant was also primarily due to defects in male mate development during meiosis I.

### Homologous chromosomal behavior is aberrant in the gametophyte development of the TE5A mutant

**Figure 4:**
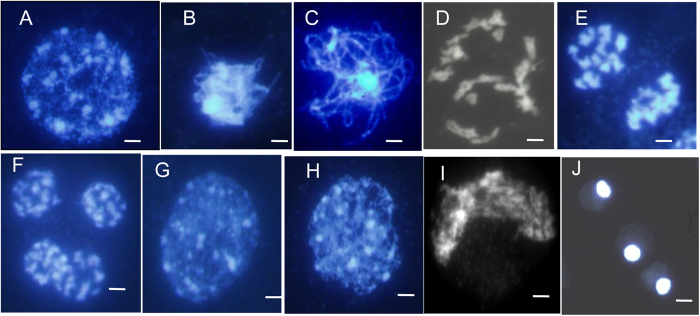
Male meiosis in fertile and sterile pollen mother cells (PMCs) prepared using the spreading technique and stained with DAPI. (**A–F**), meiosis in fertile PMCs; (**G–J**), meiosis in sterile PMCs; (**A,G**), leptotene. several bright stained chromatin centers were visible; (**B,H**), zygotene; (**C**) wild-type fertile pachytene showing full chromosome synapsis; (**D**) fertile diakinesis showing moderately condensed, unaligned bivalents; (**E**) telophase I, dyads were formed; (**F**) telophase II, tetrads were formed; (**I**) arrangement of chromosomes was disordered, and chromosomes formed the crescent-like structure; (**J**) the final arrested PMCs. Chromosomes remain together as a diffused mass. Neither meiosis I nor meiosis II is completed in these MMCs, showing meiosis being completely arrested. Scale bars = 5 μm.

To more precisely detect the gametophyte development defects in TE5A, we examined chromosome spreads from different stages of meiosis in the wild-type and the TE5A mutant. For wild-type meiosis, chromosomes condensed and appeared as thin threads in the nucleus at leptotene, and several bright stained chromatin centers were visible (Fig. 4A), which was followed by a progression of recombination and initiation of synapsis between chromosomes during the zygotene phase (Fig. 4B) and full synapsis of homologous chromosomes during the pachytene phase (Fig. 4C). At diakinesis, homologous chromosomes underwent desynapsis and further condensation, thereby forming bivalents (Fig. 4D). Homologous chromosomes moved to opposite poles with their separation from each other at telophase I (Fig. 4E). Subsequently, tetrads were formed during telophase II (Fig. 4F).

In the TE5A mutant, the PMC nuclei showed the normal leptotene phase and chromosomes also exhibited thin thread-like structures (Fig. 4G). Alterations were initially observed at the zygotene phase and the meiotic chromosomes seemed to become diffused (Fig. 4H). Thereafter, a disruption of chromosomal arrangement was observed, as indicated by the appearance of the crescent-like structure (Fig. 4I). Finally, the nuclei became more and more compact. A second meiotic division was not observed. Meanwhile, chromosomes were underwent further condensation, thereby losing its the typical chromosome morphology until fully degrading (Fig. 4J). During the entire male meiosis in the TE5A mutant, no bivalent or chromosome segregation was detected, and no dyads or tetrads were observed. We examined more than 100 meiocytes at different developmental stages in the TE5A mutant and found that they all showed the same aberrant phenotypes. These observations indicated that homologous chromosomal behavior in the male mate development was aberrant, and meiotic chromosomes might be arrested at prophase I of meiosis in the TE5A mutant.

### Homologous chromosome pairing is defective in the TE5A mutant

**Figure 5:**
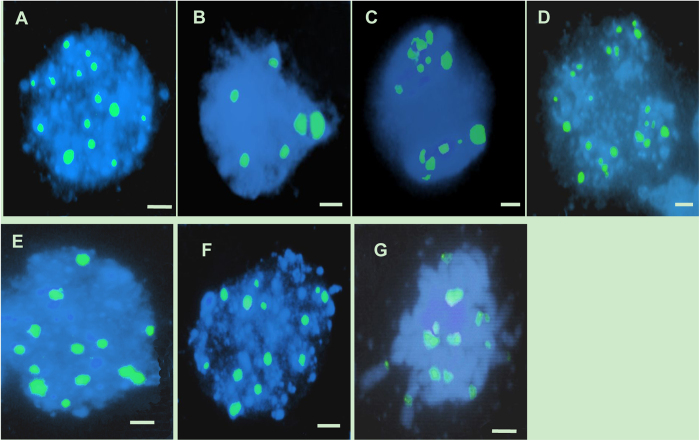
FISH analysis of meiosis in both the wild-type and the TE5A mutant. Meiotic chromosomes probed with 45S rDNA (green). Chromosomes were stained with DAPI (blue). (**A,E**), at leptotene stage, 12 to 14 45S rDNA signals were observed in the wild-type (**A**) and in the TE5A mutant (**E**), respectively; (**B,F**), at pachytene stage, six paired signals (half of the total number) were detected in the wild type (**B**), whereas 12 to 14 45S rDNA signals were still observed in the mutant (**F**,**C,D,G**) signals in half were observed in dyads (**C**) and tetrads (**D**) of the wild-type, in contrast, the number of 45S rDNA FISH foci was not halved, and unpaired 45S rDNA FISH foci were still observed in the mutant (**G**). Scale bars = 5 μm.

Further confirmation of the failure of pairing in the TE5A mutant was achieved via fluorescence *in situ* hybridization (FISH) using 45S rDNA as probe to determine the pairing status of the homologous chromosomes in PMCs during meiosis I (Fig. 5). In *Brassica napus*, there are 12 to 14 repeated 45S rDNA loci on somatic chromosomes of wild-type plants. Therefore, 12 to 14 signals should be observed in unpaired chromosomes and only six or seven in paired/synapsed chromosomes in meiosis. In both wild-type plants and the TE5A mutant, 12 to 14 45S rDNA signals were observed at leptotene (Fig. 5A,E). During early pachytene, paired signals (six signals) were detected in the wild-type (Fig. 5B), whereas 12 to 14 45S rDNA signals were also observed, thereby suggesting that a defective pairing of homologous chromosomes in the mutant (Fig. 5F). After that, the number of chromosomes was halved in the dyads (Fig. 5C) and tetrads (Fig. 5D) of the wild-type. In contrast, the number of 45S rDNA FISH foci was not halved, and unpaired 45S rDNA FISH foci were observed at the corresponding stage in the TE5A mutant with a significantly higher number of chromosomes (Fig. 5G). These results confirmed that pairing did not occur in TE5A mutant, and the arrest of prophase I might be caused by defective pairing.

### TE5A mutants have no effect on meiotic DNA replication

**Figure 6:**
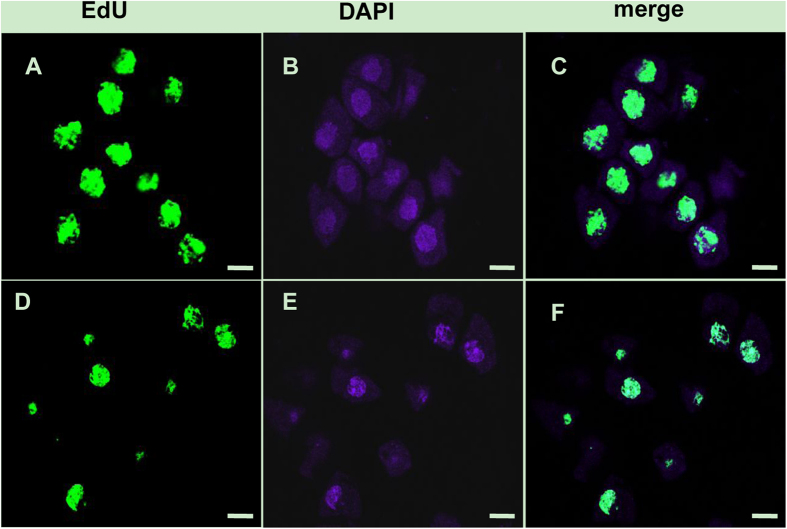
Confocal images of EdU labeled meiocytes. EdU labeling of DNA is detected by a green fluorescent signal. DNA was counterstained with DAPI (blue). EdU uptake of S-phase meiocytes revealed that TE5A mutant could succeed in completing DNA replication (**A–C**) compared with the wild-type (**D–F**). Scale bars = 5 μm.

Recently a new thymidine analog 5-ethynyl-2-deoxyuridine (EdU), was developed and utilized in investigating meiotic DNA replication. DNA is labeled through EdU uptake by S-phase meiocytes undergoing DNA replication. EdU labeling of DNA is detected with a fluorophore-tagged azide that forms a covalent bond with the terminal acetylene group on the ethynyl component of EdU. This new technology has been extensively employed in animals to study cell proliferation^41^,^42^,^43^. EdU uptake of S-phase meiocytes revealed that TE5A mutants could succeed in completing DNA replication (Fig. 6A–C) compared to the wild-type (Fig. 6D–F).

### Illumina sequencing and annotation

Genes other than that of the typical meiotic genes also play an important role in the male gamete development. During meiosis, hormone metabolism genes, serine-proteases, genes participating in cell wall biosynthesis, tapetum differentiation genes, and even polyamine biosynthesis and ribonucleases are all modulated in terms of expression^44^.

To uncover the underlying the molecular mechanism of the TE5A GMS that is associated with abnormal meiosis prophase I phase, we used young floral buds (0.8–1.0 mm in diameter, at meiotic prophase I stage) of sterile plants and fertile plants of the BC_3_ population derived from the successive backcrossing of heterozygous TE5A sterile plants with the maintainer line GY12 to perform a genome-wide high-throughput transcriptome sequencing analysis (RNA-Seq) to identify DEGs participating in the control of fertility. Table 1 shows the results of RNA-Seq and annotation of clean reads. The floral bud cDNA libraries of sterile (A1) and fertile (B1) plants were sequenced on an Illumina platform, resulting in 49,957,498 and 48,192,158 raw reads, with a total of 4,085,700,660 and 3,959,934,750 nucleotides, respectively. After filtering out reads containing unknown base N, low-quality data, and only adaptor reads, 45,396,674 and 43,999,275 reads (designated herein as “clean” reads) remained in A1 and B1 libraries, respectively, for further analysis. The full genome sequence for *B. napus* is now available. By assigning the experimental clean reads to the *B. napus* reference sequences, we observed that 29,578,223 (65.16%) and 29,370,312 (66.75%) reads matched (including perfect match and 1-bp or 2-bp mismatch) with the A1 and B1 libraries, respectively. Out of the reads mapped to the reference genes, 21,924,241 and 22,040,364 reads were uniquely matched, corresponding to 56,215 and 58,776 mapped genes for A1 and B1, respectively, and approximately 16% were matched to multiple locations, which included reads that came from repetitive sequences and were matched to highly conserved domains shared by various genes, and low-complexity reads containing poly(A) tails. The unmapped reads might be non-coding transcripts of inter-genic regions or non-annotated genes.

Sequencing saturation analysis of the two libraries was performed to assess whether the sequencing depth was of sufficient transcriptome coverage. Supplementary Figure S1 shows that with the increase of clean reads, more genes were detected. However, when the read number reached 2 million, the increase curve of the detected genes reached a plateau, which indicated that the number of acquired genes had reached saturation.

Sequence read preference of certain gene regions had a direct effect on subsequent bioinformatics analysis. Therefore, we utilized the distribution of reads on the reference genes to evaluate randomness. Supplementary Figure S2 shows that the randomness of RNA fragmentation from two samples was good, and the distributions of reads on reference genes were even.

### Gene ontology (GO) function and pathway analysis of DEGs

**Figure 7:**
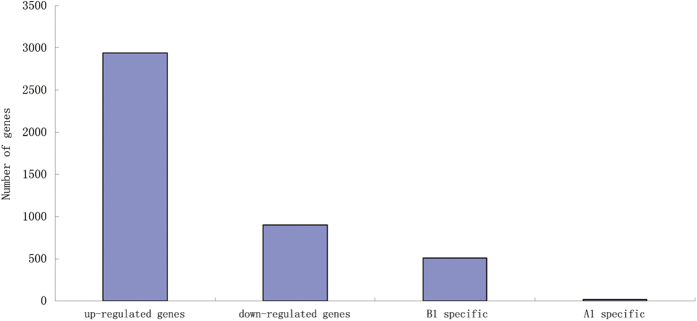
Changes in gene expression in A1 and B1 libraries. The number of upregulated and downregulated genes specific to A1 and specific to B1 are summarized.

There were 3,841 genes in *B. napus* that were detected with significant differential expression levels between A1 and B1 (Supplementary file 1). These included both 2,939 upregulated and 902 downregulated genes in B1. Of the 2,939 upregulated DEGs in B1, 511 genes were uniquely expressed in B1, which were postulated to participate in the normal development of male gametes, and only 16 of the 902 downregulated genes in B1 were specially expressed in A1, which were assumed to be involved in the abnormal development of male gametes (Fig. 7).

**Figure 8:**
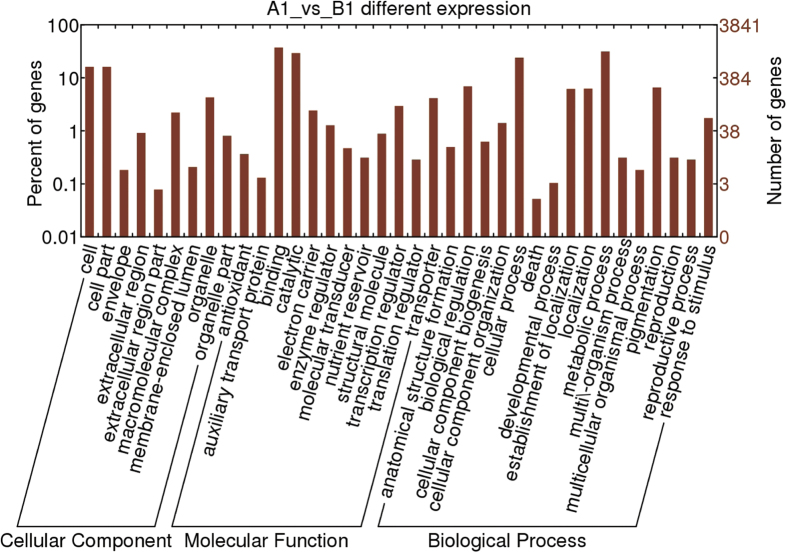
Histogram showing Gene Ontology functional analysis of DEGs. The frequency of GO terms was analyzed by using GO Slim Assignment. The y-axis and x-axis indicated the names of clusters and the ratio of each cluster, respectively.

GO is a usual standardized gene analysis classification system which categorizes genes and gene products in terms of their biological process, cellular component, and molecular function. A total of 2,339 DEGs belonging to 37 different categories were recognized (Fig. 8). For the biological processes category, genes involved in metabolism were the predominant activity (31.5%), followed by those involved in cellular processes (24%), biological regulation (6.9%), and pigmentation (6.6%). Among those with a molecular function role, 37.3% were assigned to binding, 29.2% to catalytic, and 4.1% to transporter activities. For the cellular component category, 16.1% targeted cells and cell parts, 4.3% were directed toward organelles, and 2.2% to macromolecular complexes.

To analyze the important biological functions of the DEGs, GO enrichment analyses of DEGs were performed. Supplementary file 2 shows the significantly enriched GO terms of DEGs. A comparison of the genome background with that of their function identified 7 GO terms for cellular components such as the extracellular region, external encapsulation structures, apoplasts, cell wall, cullin-Ring ubiquitin ligase complexes, liquid particles, and monolayer-surrounded lipid storage bodies. There were 27 other GO terms that were associated with transport, catabolism, transcription, and lipid localization. An additional 41 GO terms were assigned to molecular functions such as transferase, hydrolase, and enzyme inhibitor activities. Further analysis led to the assignment of 7 DEGs to the histone acetyl transferases Fna019097, Fna026265, Fna006674, Fna008350, Fna060150, Fna009968, and Fna079142, which were all upregulated in B1. Histone acetyltransferases acetylate specific lysine residues that are mainly located at the amino terminal tail of histones^45^, thereby participating in the recruitment of *trans*-acting regulatory factors^46^,^47^, chromosome segregation, and the establishment of chromatin boundaries^48^,^49^.

Genes usually play a role in special biological functions by interacting with each other. Pathway-based analysis helps to further elucidate the biological functions of DEGs. KEGG is the major public pathway-related database^50^. Pathway enrichment analysis identifies significantly enriched metabolic pathways or signal transduction pathways in DEGs relative to the background of the entire genome. In the present study, the enriched pathways included flavonoid biosynthesis, linoleic acid metabolism, carotenoid biosynthesis, alpha-linolenic acid metabolism, phenylpropanoid biosynthesis, cyanoamino acid metabolism, plant circadian rhythm, and glucosinolate biosynthesis (Supplementary file 3). Further research on these genes that were involved in certain pathways would help to elucidate the mechanisms underlying GMS in TE5A mutants.

### DEGs are involved in homologous chromosome behavior during meiosis

TE5A meiocytes exhibited abnormal chromosome behavior during meiosis (Fig. 4), prompting us to further investigate whether meiotic gene expression was affected. Prophase I of meiosis is characteristic of homologous chromosome pairing, cohesion, recombination, and synapsis^51^. Three upregulated DEGs (Fna021329, Fna087283, and Fna002253) encoding helicases were identified in B1 (Table 2). RECQ HELICASES are involved in DNA recombination and replication during later phases of DSB repair and are possibly related to branch migration at the Holliday junction^52^. Eight DEGs (Fna064688, Fna018802, Fna063085, Fna048643, Fna072157, Fna002765, Fna044947, and Fna086175) encoding topoisomerases (including DNA topoisomerase III and DNA topoisomerase I) were observed, of which five were upregulated in B1 (Table 2). In the budding yeast, *SPO11* is essential for the initiation of meiosis recombination^53^, catalyzing DSB formation by using the activity of a topoisomerase-like enzyme^54^. Two additional *Spo11* genes, namely, *AtSPO11-2* and *AtSPO11-3*, have been identified and were reported to interact with a subunit B of archaebacterial-type topoisomerase 6 in *A. thaliana*^55^. These results thus showed that DNA recombination might be affected in A1.

In addition, specific sequence distribution, chromosome morphology, and the chromosome condensation patterns that give a chromosome a characteristic shape may be involved directly or indirectly in homologous chromosome recognition^56^. *REC8/RAD21* is involved in chromosome pairing, condensation, and cohesion of sister chromatids in mitosis^57^,^58^,^59^. In the present study, two DEGs encoding *REC8/RAD21* (Fna075597 and Fna016698) were upregulated in B1 (Table 2). Four other DEGs involved in the regulation of chromosome condensation (Fna070368, Fna077294, Fna054687, and Fna058087) were also upregulated in B1 (Table 2), indicating that chromosome condensation associated with chromosome pairing was affected in A1.

### DEGs are involved in the meiotic cycle control

Meiosis-specific modulatory mechanisms have been developed for the successive separation of homologous chromosomes during meiosis I and separation of sister chromatids in meiosis II. Oscillations in CDK activity play an important role in meiosis progression^60^,^61^. In meiosis, exit from anaphase depends on loss of cyclin-dependent kinase (CDK) activity, whereas entry into the M-phase requires high activity^60^. Morphological analysis of gametogenesis and male meiosis has indicated that loss of CDKA;1 resulted in complete sterility^62^,^63^. The TE5A mutants were infertile due to meiotic arrest at prophase I. One gene (Fna032795) encoding cyclin-dependent kinase C;1 (CDKC;1) and another gene encoding Cdk-activating kinase assembly factor (MAT1) (Fna008591) were upregulated in B1 (Table 3). Seven genes encoding CDK inhibitors (Fna043714, Fna060576, Fna040927, Fna008334, Fna063948, Fna018615, and Fna060445) were also upregulated in B1 (Table 3). These results indicated that an oscillation failure of CDK activity regulation might be associated with prophase I arrest in TE5A mutants.

CDK activity is regulated at various levels, and an important determinant of CDK is the type and amount of available cyclin partners^64^. For the detected DEGs in the present study, 108 cyclin genes showed significant differential expression levels, most of which contain the F-box domain (Supplementary file 4). These included 101 upregulated and 7 downregulated genes in B1. Of the 101 upregulated genes encoding cyclin, 57 genes were uniquely expressed in B1. However, these cyclins has not yet been functionally characterized in meiosis. Cyclin-CDKs form different complexes that control the progression of the meiotic cell cycle. Additional studies aimed to fully elaborate the modulatory network underlying meiosis progression in TE5A are thus required.

### Dynamic expression changes of candidate DEGs at different anther developmental stages

For *Brassica napus*, the relationship between bud lengths and anther developmental stages has been identified. Bud lengths <0.5, 0.5–1, 1–1.5, and 1.5–2.0 mm represented the PMC stag, the meiosis stage, the tetrad stage, and microspore release stage, respectively^65^. To further investigate the expression levels of some candidate genes identified by RNA-Seq, total RNA was isolated from buds from lines A1 (sterile) and B1 (fertile) at four specific stages (<0.5, 0.5–1, 1–1.5, and 1.5–2.0 mm) and analyzed by using real-time PCR, respectively. All primers are listed in Supplemental Table S1.

**Figure 9:**
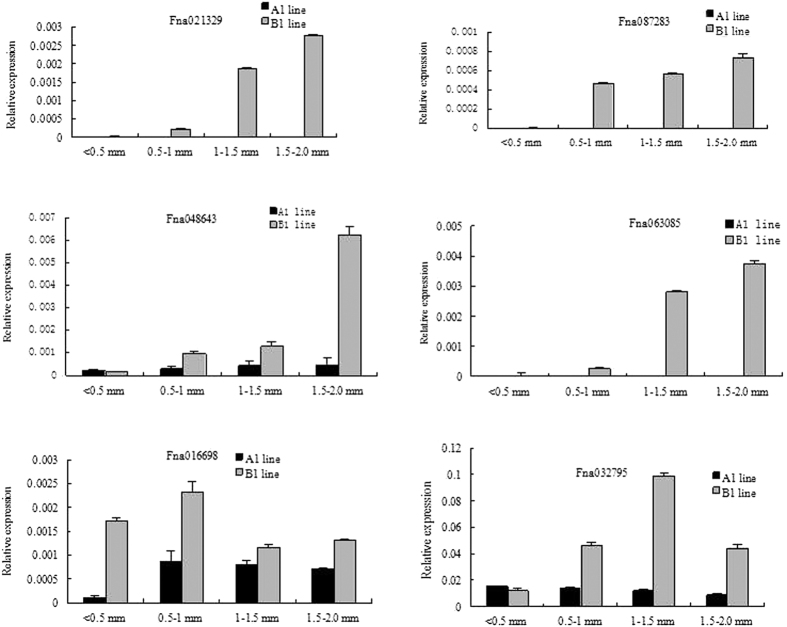
Dynamic expression changes of candidate DEGs at different developmental stages based on real-time PCR. A total of six DEGs, Fna021329, Fna087283, Fna048643, Fna063085, Fna016698, and Fna032795 were selected for further analysis at different anther development stages in the A1 and B1 lines. Relative levels of genes in real-time PCR are presented by 2^−ΔΔCT^.

RECQ HELICASES are involved in DNA recombination during later phases of DSB repair and are possibly related to branch migration at the Holliday junction^52^. In the present study, the differential expression of two DEGs encoding helicase, Fna021329, and Fna087283 were examined during above four anther developmental stages in A1 (sterile) and B1 (fertile) lines by using real-time PCR. Both Fna021329 and Fna087283 were not expressed during the four stages in line A1, whereas these were expressed at the PMC stage, and further upregulated at the meiosis, in the tetrad, and the microspore stage in the B1 line (Fig. 9).

Topoisomerases are involved in various cell processes, including transcription, recombination, chromatin remodeling, and DNA replication by cutting DNA strands in a irreversible manner, which in turn release impending topological forces. Two DEGs encoding topoisomerases, namely Fna048643 and Fna063085, were analyzed by real-time PCR. The expression of Fna048643 was lower at various anther development stages in line A1 compared to that in the B1 line, and its expression markedly increased after the PMC stage in line B1 (Fig. 9). The expression of Fna063085 was not detected in line A1 at various anther development stages, whereas its expression level was high at the tetrad and the microspore stages in the B1 line (Fig. 9).

Cohesins are conserved proteins that have been implicated in the cohesion of sister chromatids in meiosis and mitosis and are responsible for DSB repair and meiotic recombination. In the present study, the expression of Fna016698, which encodes members of the *REC8/RAD21* cohesin family, was suppressed at the PMC and the meiosis stages in the A1 line, whereas at the tetrad and the microspore stages, its expression showed little detectable differences between the A1 and B1 lines (Fig. 9).

Oscillations in CDK activity play an important role in meiosis progression^61^. In the present study, one gene, Fna032795, which encodes cyclin-dependent kinase C;1 (CDKC;1), was upregulated at the meiosis stage, the tetrad stage, and the microspore stage in the B1 line (Fig. 9). In addition, differential expression of Fna032795 was observed at the PMC stage, the meiosis stage, the tetrad stage, and the microspore stage in the B1 line, whereas no obvious changes of expression were detected at the four stages in the A1 line (Fig. 9).

### TE5A mutants as a model for GMS

**Figure 10:**
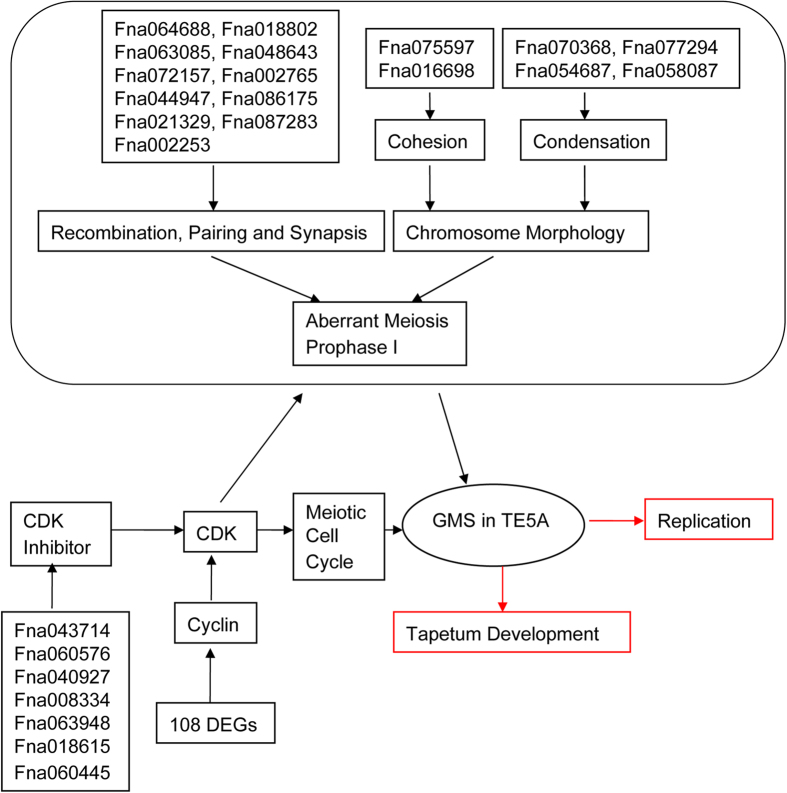
Model of GMS in TE5A. The network of GMS in TE5A based on the analysis of cytological observation and RNA-seq. Red boxes indicate no difference between the wild-type and the TE5A mutant.

Cytological observation and the differential gene expression data generated from RNA-Seq indicate that TE5A mutants can be utilized as a model for GMS (Fig. 10). Successful male development are related to various important development events, including cell differentiation, meristem specification, communication among cells, mitosis and meiosis^51^,^60^. Paraffin-cross sections of anthers showed that male gamete development was arrested at the first meiotic division stage, and TEM analysis of cross sections of anthers indicated that PMCs were arrested at prophase I, which eventually resulted in the degeneration of meiotic PMCs in TE5A. Meiotic prophase I in the wild-type is characterized by pairing, chromosome cohesion, and recombination^51^. Chromosome spreads and FISH showed that homologous chromosomes could not pair, synapse, condense, and form bivalents in the TE5A mutant. RNA-Seq further uncovered DEGs associated with the defects observed during TE5A mutant gametogenesis, including DEGs that were involved in homologous chromosome behavior and the cycle control during meiosis.

## Discussion

The present study examined the cytological defects of anther development in the TE5A mutant. The TE5A mutant showed abnormal homologous chromosomal behavior during prophase I. In addition, EdU uptake of S-phase meiocytes revealed that TE5A mutants could accomplish DNA replication. To gain a deeper insight into the GMS of TE5A mutant, the RNA-Seq approach was used to identify DEGs between the sterile mutant (A1) and the fertile wild-type (B1). DEGs responsible for recombination, pairing and meiotic cycle control were identified. Finally dynamic expression changes of candidate DEGs at different developmental stages of anthers were analyzed by real-time PCR in both the TE5A mutant and the wild-type. Because of without biological replicates, additional studies that would verify the candidate DEGs are warranted.

### Defects in male gamete development of the TE5A mutant

The TE5A mutant showed normal floral morphology and architecture as that in the wild-type, except for the withered anthers with no pollen (Fig. 1). Successful male reproductive development within the anther, includes a number of critical developmental events such as meristem specification, cell differentiation, cell-to-cell communication, meiosis and mitosis^51^,^60^. Analysis of paraffin-cross sections of anthers and TEM analysis showed that the male gamete development was arrested at meiosis I. Eventually, meiotic PMCs underwent degeneration, thereby resulting in an empty locule, and no pollen grains were generated in the TE5A mutant (Figs 2 and 3). Chromosome spreads and FISH showed that homologous chromosomes could not pair, synapse, condense, and form bivalents in TE5A mutant, and meiotic chromosomes might have been arrested at prophase I of meiosis in the TE5A mutant (Figs 4 and 5).

Prophase I which is the longest and most complex phase of meiosis, is a vital stage that ensures the correct completion of the meiotic program. A series of chromosome dynamics associated events during meiotic prophase I such as chromosomal reorganization and condensation, establishment of meiotic-specific chromosome structure, homologous chromosome pairing, and dynamic chromosome movements are closely integrated and finely controlled temporally and spatially to contribute to meiosis^66^,^67^,^68^,^69^,^70^,^71^. At the end of meiotic prophase I bivalents are present. These bivalents consist of highly condensed paired homologous chromosomes joined at chiasmata, which are the physical sites of crossover between homologous chromosomes and are only established if pairing and recombination occur normally. There are many mutations affecting meiotic prophase I that often result in 10 univalents instead of bivalents in Arabidopsis^30^,^57^,^58^,^64^,^65^,^72^,^73^. This can result in a random unbalanced segregation of univalents in meiosis. In most cases the cells produced are aneuploid and abort during development. However, in the present study, aberrant PMCs were eventually degraded, thereby resulting in complete sterility. It has been postulated that this result may have been caused by a defect in a cell cycle-checkpoint.

### DEGs associated with homologous chromosomal behaviour during meiosis

Meiosis is a specialized cell division with two successive rounds of chromosome segregation following a single round of DNA replication, by which haploid cells are generated^74^. Chromosome dynamics during meiotic prophase I are associated with a series of major events. In early meiosis, adoption of the meiosis specific chromosome structure by chromatin condensation in the leptotene stage is one of the key processes in meiotic prophase I^70^,^71^. Homologous chromosome pairing has been shown to be tightly linked to the progression of meiotic recombination^69^. Homologous recombination is essential for acquiring physical juxtaposition between homologous chromosomes^75^. The following homologous chromosome pairing is colsely tightly linked to the progression of meiotic recombination^69^. Synapsis and recombination ensure the establishment of chiasmata that hold homologous chromosomes together allowing their correct segregation. Several genes have already been identified to play essential roles in meiotic recombination particularly in the establishment of their functions, interactions, and timing, ultimately facilitating in building a comprehensive network that is related to the recombination pathway of meiosis^76^,^77^,^78^. In general, recombination genes are highly conserved and direct homologues have been identified in various organisms.

TE5A meiocytes exhibited abnormal chromosome behavior during meiosis (Figs 4 and 5). In this study, 3 DEGs encoding RECQ HELICASES, 5 out of 8 DEGs encoding topoisomerase, and 6 DEGs associated with chromosome morphology and condensation, including two *REC8/RAD21* cohesin family members were all upregulated in B1 (Table 2). RECQ HELICASES are involved in DNA recombination during later phases of DSB repair and are possibly related to branch migration at the Holliday junction^52^. In *Saccharomyces cerevisiae*, *Sgs1*, a the BLM helicase ortholog, is involved in normal meiosis recombination, other than its activity of restricting abnormal recombination intermediates^79^. In addition, Sgs1 helicase is also essential for meiotic recombination in a protist that does not develop synaptonemal complexes^80^. Topoisomerases are involved in DNA metabolism and the DNA supercoiling steady-state level in eukaryotes and prokaryotes^81^,^82^. Topoisomerases are involved in various cell processes, including transcription, recombination, chromatin remodeling, and DNA replication by cutting DNA strands in an invertible manner, which in turn release impending topological forces. Topoisomerase II is essential for chromosome segregation in meiosis I, thus helping the separation of recombined chromosomes^83^. In *Arabidopsis*, the top3α-2 mutant shows stunted growth and complete sterility. Specific sequence distribution, chromosome morphology, and the chromosome condensation patterns that give a chromosome a characteristic shape may be directly or indirectly involved in the homologous chromosome recognition^56^. The *DIF1* gene of *Arabidopsis*, one member of the *REC8/RAD21* cohesin family, is essential for meiosis chromosome separation. Mutations in the *DIF1* gene mainly lead to female and male sterility^57^. Two major cohesins, REC8 and RAD21, function in meiosis of *S. pombe*. RAD21 interacts with telomeres, whereas REC8 mainly associates with centromeres^84^.

During meiosis in most organisms, production of haploid gametes is accompanied by frequent recombination between homologous parental chromosomes. Recombination occurs after meiotic DNA replication. Borde *et al*.^53^ have clearly shown that DNA replication is required for DSBs as a safety check to ensure that breakage is not induced before sister chromatids are available for repair, in case corresponding sequences on homologous chromosomes could not be found^85^. In the yeast *Saccharomyces cerevisiae*, blocking meiotic replication has been shown to prevent recombination^86^,^87^,^88^. S-phase is the important portion meiotic cell cycle when DNA replicates. Systematic studies have revealed that the S-phase that precedes meiosis prophase I is required for double-strand break (DSB) formation^89^. EdU uptake of S-phase meiocytes revealed that TE5A mutants could succeed in completing DNA replication (Fig. 6) compared to the wild-type.

### DEGs associated with the meiotic cycle control

Meiosis has two consecutive rounds of chromosome division and only one DNA replication compared to mitosis^90^. Meiosis specific events are closely integrated and finely controlled temporally and spatially^66^,^67^,^68^. The oscillations in CDK activity can coordinate the various events of the cell cycle and develop a unidirectional process during the cell cycle through preventing untimely the nuclear DNA re-replication before mitosis. Although meiosis-specific modulatory mechanisms were developed to accomplish successive separation of homologous chromosomes during meiosis I and separation of sister chromatids in meiosis II, meiosis and mitosis share important principles controlling cell cycle. Exit from anaphase depends on a loss of cyclin-dependent kinase (CDK) activity, whereas entry into the M-phase in meiosis requires a high activity. The most noticeable meiotic event is that DNA replication is suppressed after meiosis I. This is accomplished by precise modulation of CDK activity which decreases to a certain level that allows spindle rearrangement and chromosome decondensation and is still adequate to suppress the assembly of pre-replicative complexes^60^.

Early experiments using temperature-sensitive Cdc28/Cdc2 yeast mutants and *Xenopus* oocytes have shown that CDKs play a central role in meiosis progression, similar to that observed in mitosis^61^. Five central CDKs, CDKA;1, CDKB1;1, CDKB2;2, CDKB1;2, and CDKB2;1, have already been reported so far in *Arabidopsis*^91^. *Arabidopsis* CDKA;1 demonstrates the highest similarity with Cdk2 and Cdk1 in animal species^92^. CDKA;1 mutants are completely sterile, and morphological analysis of gametogenesis and male meiosis has indicated that it plays an important role during meiosis^62^,^63^. CDKA;1 apparently plays an essential role throughout meiosis; A high kinase activity of seems to be crucial in preventing premature meiosis I exit^92^. In the present study, one gene (Fna032795) encoding cyclin-dependent kinase C;1 (CDKC;1) and another gene encoding Cdk-activating kinase assembly factor (MAT1) (Fna008591) were upregulated in B1 (Table 3). However, it was puzzling that 7 genes encoding CDK inhibitor (Fna043714, Fna060576, Fna040927, Fna008334, Fna063948, Fna018615, and Fna060445) were also upregulated in B1 (Table 3).

CDK activity is regulated at various levels, and an important determinant of CDK is the type and amount of available cyclin partners^93^. Cyclins are common CDK activity activators and regulators of the cell cycle^94^. Prediction analysis of the *Arabidopsis* genome indicates that there are approximately 21 B- and A-type cyclins^95^, however, our understanding of the function of the majority of cyclins in meiosis is limited. Only two cyclins have already been reported to play an important role in meiosis, namely, SDS^96^ and TAM^97^. For the detected DEGs in the present study, 108 cyclin genes showed significant differential expression levels (Supplementary file 4). However, these cyclins has not yet been functionally characterized in meiosis. Cyclin-CDKs form different complexes that control the progression of the meiotic cell cycle. Altered expression of these cyclins in TE5A is thus required to further examine its activity and function. The results of the present study indicated that the obaerved prophase I arrest in TE5A mutants was likely caused by an oscillation failure of CDK activity regulation. More studies that would elucidate the regulatory network underlying meiotic cell cycle progression in TE5A are warranted.

## Conclusions

TE5A is a newly bred DGMS line that is characterized by abnormal meiotic chromosome behavior. In the present study, paraffin-cross sections of anthers showed that male gamete development was arrested at the first meiotic division stage, and TEM analysis of cross-sections of anthers showed that PMCs were arrested at prophase I. EdU uptake of S-phase meiocytes revealed that TE5A mutant could accomplish DNA replication, and chromosome spreads and FISH showed that homologous chromosomes could not pair, synapse, condense, and form bivalents in TE5A mutants. To elucidate the underlying molecular mechanism of the TE5A GMS that is associated with abnormal meiosis prophase I phase, RNA-Seq was performed, identifying DEGs between floral buds of fertile and sterile plants. A total of 3,841 DEGs were detected, some of which were associated with homologous chromosome behavior and cell cycle control during meiosis. These DEGs represented a set of potential candidate genes associated with GMS in the TE5A. Finally dynamic changes in the expression of candidate DEGs were then detected at different anther developmental stages. The present study provided a global assessment of the differences between sterile plants and its near-isogenic fertile plants, as well as identified new fertility-associated genes, which may lie a strong foundation for future research on GMS in the TE5A line. Although we have identified a number of DEGs, most of which are due to secondary and/or tertiary actions from the gene conferring male fertility. If the MS (male sterile) gene is mapped, we should compare these DEGs that are mapped to the MS locus to see which genes may have sequence variation. Only these genes that are differentially expressed with sequence variation surrounding the MS locus may be considered the candidate genes for a further analysis.

## Methods

### Plant materials

To directly assess fertility, plants were grown in Xining (a spring oilseed rape area in Qinghai Province, Northwest China), in which the temperature is >20 °C during florescence, and the TE5A line is completely male sterile. One heterozygous TE5A sterile plant, as the female parent, was crossed with the temporary maintainer, GY12, resulting in a fertility segregation F_1_ population. Sterile F_1_ plants were backcrossed to GY12 to produce the BC_1_ population. The BC_3_ population was obtained by successive backcrossing of sterile plants of the BC population to GY12. Sterile and fertile plants with 1:1 fertility segregation ratio derived from the BC3 population were near-isogenic lines (NILs), differing only in the fertility trait. Fertility of the plants was determined during flowering. For RNA-Seq, a total of 0.5 g young floral buds (0.5–1.0 mm in diameter) was collected and stored at 80 °C until analysis.

### Light microscopy and TEM analyses

Morphological observations of paraffin-cross sections and DAPI staining were performed according to the described methods^98^. Micrographs of fluorescence of chromosomes were captured by the Nikon Eclipse 80i fluorescence microscope, equipped with the appropriate filter and illuminated by using ultraviolet light. Fresh anthers of the wild-type and 7365A mutant plants at various developmental stages were fixed in 2.5% (w/v) glutaraldehyde in 0.1 M phosphate buffer (pH 7.4) for TEM analysis. The following procedures were performed as previously described^99^.

### RNA-Seq

Total RNA of young floral buds (0.8–1.0 mm in diameter) from five sterile and fertile individual plants, respectively, was extracted using the TRIzol reagent (Invitrogen, CA, USA) and bulked as pools. Total RNA samples were, first, treated with DNase I to eliminate DNA contamination. Then, the mRNA samples were gathered by utilizing magnetic beads. The mRNA samples, which were dissolved in the appropriate buffer, were fragmented into short segments (about 200 bp in length). cDNA was then synthesized by utilizing random hexamer primers and was purified by using magnetic beads. The reparation of DNA ends and 3′-end adenine (A) addition were then conducted. Finally, adaptors were linked to the fragments, and the fragments were amplified by PCR. The library products were then sequenced using the Illumina HiSeqTM 2000. Clean reads were matched to the transcriptome sequences of *B. napus* (http://www.oilcrops.info/)^100^ using SOAP aligner/SOAP2^101^. Less than 2 mismatches were allowed in each alignment. Unambiguous clean tags were obtained after filtering clean tags matched to multiple genes. Unambiguous clean tags of each gene was then counted and normalized to the number of transcripts per million tags (TPM)^102^. The data used in this study were deposited to NCBI’s Sequence Read Archive (SRA) and are accessible through SRA Series accession number SRP068170 (http://www.ncbi.nlm.nih.gov/sra?term=SRP068170).

### Screening and analysis of DEGs

The expression level of each gene was determined by the number of reads that was uniquely mapped to the specific gene and the total number of uniquely mapped reads in the sample by using the reads per kb per million reads (RPKM) method^103^. DEGs between the two samples were identified by a rigorous algorithm^104^,^105^. The value of |log_2_ratio| ≥ 1 and FDR ≤ 0.001 were utilized as thresholds to determine the significant differences in transcript abundance. DEGs were also identified by more stringent standards with greater fold-change and smaller FDR values. GO functional classification of DEGs was performed using software WEGO. KEGG analysis (http://www.genome.jp/kegg) was also carried out based on the method of Yuan *et al*.^106^. Enrichment analyses of GO and pathway used the hypergeometric test^107^,^108^. The GO term (P ≤ 0.05) was defined as a significantly DEG-enriched GO term. The pathways with a Q value of ≤0.05 were defined as those with significant DEGs.

### Real-time PCR analysis

Real-time PCR analysis was used to verify the DEG results. The RNA samples used for the qRT-PCR verification assays were the same as that employed in the RNA-Seq experiments. Total RNAs isolated from four bud stages (<0.5, 0.5–1, 1–1.5, and 1.5–2.0 mm) for the A1 (sterile) and B1 (fertile) lines were used in real-time PCR analysis to further investigate the expression levels of specific candidate genes identified in the RNA-seq data. qRT-PCR was conducted according to the TaKaRa manufacturer specifications on an iQ™ 5 Multicolor Real-time PCR Detection System (Bio-RAD, USA). The *B. napus* actin gene was used as an internal control to normalize the expression data, and the relative expression levels of genes were calculated by using 2^−ΔΔCT^.

### DNA replication analysis

DNA replication analysis was performed according to the described methods^109^. Stems of young inflorescences were cut and submerged in 10 mM EdU labeling solution from a Click-IT assay kit (Invitrogen, CA, USA). These inflorescences were placed at 21 °C for 8 h. Labeled inflorescence were fixed through 3.7% formaldehyde and then placed in EdU Click-It color reaction cocktail for one hour according to manufacturer’s instructions (Invitrogen). Fluorescence Images were captured using a confocal laser microscope.

### FISH analysis

Methods for FISH had been described previously^110^. In detail, anthers with pollen mother cells at meiotic stages were fixed, and slides were prepared. The probes used for FISH was 45S rDNA from clone pTa71^111^, which was labeled with digoxigenin-11-dUTP by nick translation (Roche). Dioxigenin-labeled DNA were detected with Fluorescein isothiocyanate conjugated (FITC) anti-digoxigenin antibodies (Roche). Slides were counterstained with DAPI (1 mg/mL). Fluorescence images were captured using a confocal laser microscope. The sites of antidigoxigenin–fluorescein-detected probes were green, and DAPI staining was blue. The final images were merged using the Adobe Photoshop 5.0 software.

## Additional Information

**How to cite this article**: Yan, X. *et al*. Aberrant Meiotic Prophase I Leads to Genic Male Sterility in the Novel TE5A Mutant of *Brassica napus*. *Sci. Rep*. **6**, 33955; doi: 10.1038/srep33955 (2016).

## Supplementary Material

Supplementary Information

Supplementary File 1

Supplementary File 2

Supplementary File 3

Supplementary File 4

## Figures and Tables

**Table 1:** Summary statistics of RNA-Seq and annotation of clean reads against reference sequences of *Brassica napus*.

Sample ID	Raw reads	Clean reads	Total base pairs	Total mapped reads	Perfect match	Mismatch	Unique match	Multip -osition match	Total unmapped genes	Mapped genes
A1	49,957,498	45,396,674	4,085,700,660	29,578,223	20,400,456	9,177,767	21,924,241	7,653,982	15,818,451	56,215
B1	48,192,158	43,999,275	3,959,934,750	29,370,312	20,122,638	9,247,674	22,040,364	7,329,948	14,628,963	58,776

Mismatch < = 2 bp.

**Table 2:** DEGs involved in homologous chromosome behavior during meiosis.

Gene ID	log2 Ratio (B1/A1)	Up/Down (B1/A1)	Funtional annotation
Recombination helicase			
Fna021329	9.212104581	Up	DNA recombination; ATP-dependent helicase activity
Fna087283	4.107856066	Up	DNA recombination; ATP-dependent helicase activity
Fna002253	1.259859159	Up	helicase activity; DNA repair and recombination protein RAD54
Topoisomerase			
Fna064688	9.413224204	Up	DNA topoisomerase II, DNA gyrase subunit B
Fna018802	8.983925591	Up	DNA topoisomerase III
Fna063085	5.607088692	Up	DNA topoisomerase I
Fna048643	4.484231944	Up	DNA topoisomerase I
Fna072157	3.992378848	Up	DNA topoisomerase I
Fna002765	−1.48155234	Down	DNA topoisomerase III
Fna044947	−1.30118435	Down	DNA topoisomerase, nuclear transcription factor Y, beta
Fna086175	−1.22206482	Down	DNA topoisomerase, nuclear transcription factor Y, beta
Chromosome condensation			
Fna070368	3.301131554	Up	Regulator of chromosome condensation (RCC1)-like protein
Fna077294	1.918378267	Up	Regulator of chromosome condensation, RCC1
Fna054687	1.219447757	Up	Regulator of chromosome condensation, RCC1
Fna058087	1.21914971	Up	Regulator of chromosome condensation, RCC1
Rad21/Rec8-like protein			
Fna075597	1.689451715	Up	cohesin complex subunit SCC1
Fna016698	1.374249483	Up	cohesin complex subunit SCC1

**Table 3:** Some DEGS involved in Cell cycle.

Gene ID	log2 Ratio(B1/A1)	Up-Down (B1/A1)	Funtional annotation
Fna043714	11.86690003	Up	CDK inhibitor
Fna060576	4.812557811	Up	CDK inhibitor
Fna040927	4.79973377	Up	CDK inhibitor
Fna008334	3.636235038	Up	CDK inhibitor
Fna063948	2.833681102	Up	ankyrin repeat protein nuc-2, putative; CDK inhibitor PHO81
Fna008591	2.259165389	Up	Cdk-activating kinase assembly factor (MAT1)
Fna018615	2.005052678	Up	CDK inhibitor
Fna060445	1.600061425	Up	CDK inhibitor
Fna032795	1.029090626	Up	Cyclin-dependent kinase C;1 (CDKC;1)
