# Supplementary material for: Aberrant Meiotic Prophase I Leads to Genic Male Sterility in the Novel TE5A Mutant of Brassica napus
Source: Sci Rep. 2016 Sep 27;6:33955. doi: 10.1038/srep33955 (PMC5037387; doi:10.1038/srep33955)
Supplement: Supplementary Information [file srep33955-s1.pdf]

Aberrant Meiotic Prophase I Leads to Genic Male Sterility in the Novel TE5A Mutant of *Brassica napus*

Xiaohong Yan[†], Xinhua Zeng[†], Shasha Wang, Keqi Li, Rong Yuan, Hongfei Gao, Junling Luo, Fang Liu, Yuhua Wu, Yunjing Li, Li Zhu and Gang Wu*

Oil Crops Research Institute of the Chinese Academy of Agricultural Sciences/Key Laboratory of Biology and Genetic Improvement of Oil Crops, Ministry of Agriculture, Wuhan 430062, China

* Correspondence: wugang@cass.cn

† Contributed equally

The supplementary information includes two figures, one table, and four large datasets as separate Excel documents. Figure S1 shows sequencing saturation analysis of the two libraries of A1 and B1. Figure S2 shows distributions of clean reads from the A1 and B1 samples on reference genes. Table S1 provides the primer information used for qRT-PCR analysis. Supplementary dataset 1 shows DEGs of B. napus between A1 and B1. Supplementary dataset 2 shows Significantly enriched GO terms of DEGs. Supplementary dataset 3 shows Significantly enriched pathways of DEGs. Supplementary dataset 4 shows Cyclin genes of DEGs

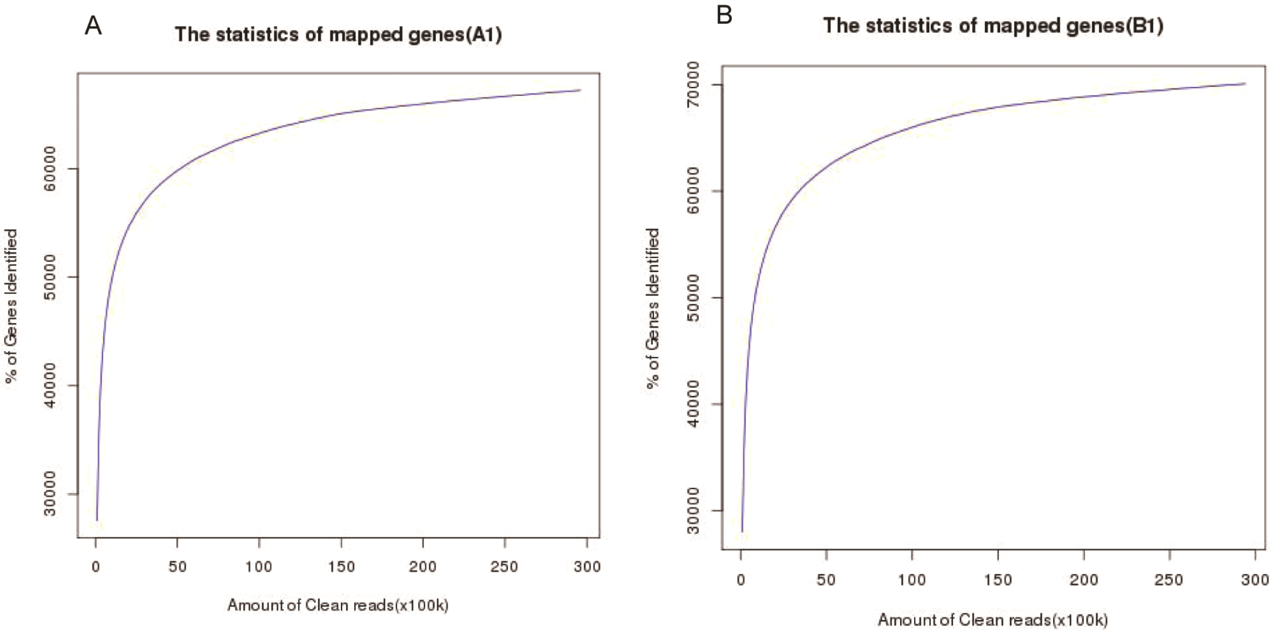

Figure S1. Sequencing saturation analysis of the two libraries of A1 and B1. The number of detected genes was enhanced as the sequencing amount (total number of clean reads) increased. A, Clean reads of A1 mapped to *B. napus*; B, Clean reads of B1 mapped to *B. napus.*

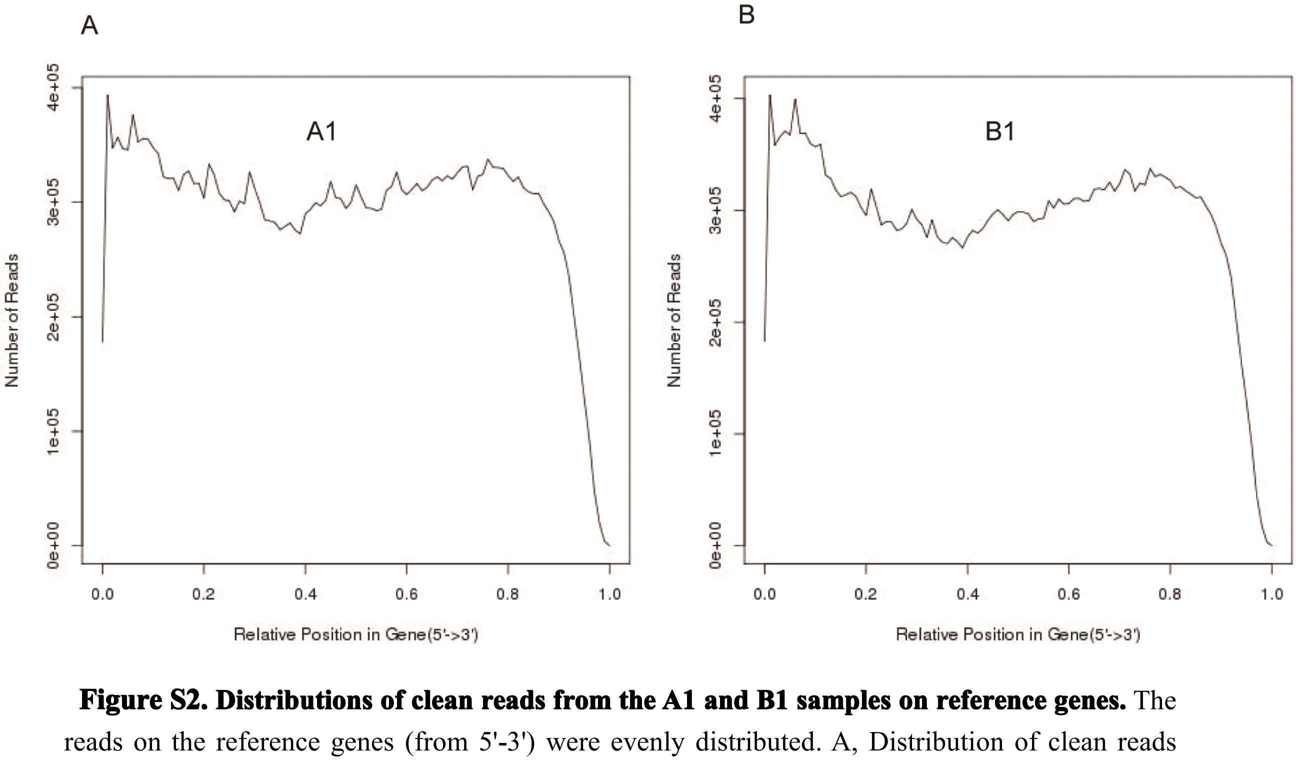

Figure S2. Distributions of clean reads from the A1 and B1 samples on reference genes. The reads on the reference genes (from 5'-3') were evenly distributed. A, Distribution of clean reads from A1 on the reference genes; B, Distribution of clean reads from B1 on the reference genes.

Table S1 Primers used in qRT-PCR analysis

Amplified genes	Primer sequences
actin	F:CTGGAATTGCTGACCGTATGAG<br>R:ATCTGTTGGAAAGTGCTGAGGG
Fna021329	F: CTTCACCGAGTCCGTAGCAT<br>R: TCCGACAGGACGAAAGAAAC
Fna087283	F: CATCAGTCCTACGACGTTCA<br>R: CTCCTTCTTGTCATTCACCC
Fna048643	F: TGTGGGCAGTTCTTGATGGC<br>R: TTCACCAGGGGTAGGGCATT
Fna063085	F: GATGCTCCTATCCCTGAATG<br>R: CTCGTACTTATCCTTGTCGC
Fna016698	F: ATGCTGCTGCTCACGGTTTA<br>R: CAAGGTTAGGGATACTGGGT
Fna032795	F: GTTGACATTTGGTCGGTTGC<br>R: CTTCAGAGGCAGTGATGGTT
